# Rapamycin inhibits proliferation and induces autophagy in human neuroblastoma cells

**DOI:** 10.1042/BSR20181822

**Published:** 2018-11-30

**Authors:** Xiaokun Lin, Lei Han, Jialei Weng, Kelai Wang, Tongke Chen

**Affiliations:** 1 Department of Pediatric Surgery, Qilu Hospital of Shandong University, Jinan 250012, Shandong Province, China; 2 Department of Pediatric Surgery, The Second Affiliated Hospital and Yuying Children’s Hospital of Wenzhou Medical University, Wenzhou 325027, Zhejiang Province, China; 3 The Second School of Medicine, Wenzhou Medical University, Wenzhou 325000, Zhejiang Province, China; 4 Laboratory Animal Center, Wenzhou Medical University, Wenzhou 325000, Zhejiang Province, China

**Keywords:** Autophagy, Neuroblastoma, Proliferation, Rapamycin

## Abstract

**Objective** To investigate the effect of Rapamycin on proliferation and autophagy in human neuroblastoma (NB) cell lines and to elucidate the possible mechanism. **Methods** NB cells were treated with different concentrations of Rapamycin. Cell counting kit-8 (CCK-8) was used to measure proliferation, and flow cytometry (FCM) was used to analyze the cell cycle. EM was used to observe cell morphological changes. Western blotting (WB) was performed to detect the expression of Beclin-1, LC3-I/II, P62, mammalian target of Rapamycin (mTOR), and p-mTOR. **Results** Rapamycin inhibited the spread of NB cells in a dose- and time-dependent manner and arrested the cell cycle at the G_0_/G_1_ phase. EM showed autophagosomes in NB cells treated with Rapamycin. The WB results showed that the expression levels of Beclin-1 and LC3-II/LC3-I were significantly elevated in NB cells treated with Rapamycin, while the expression levels of P62, mTOR, and p-mTOR proteins were significantly reduced compared with the control cells (*P*<0.05). **Conclusion** Rapamycin inhibits cell proliferation and induces autophagy in human NB cell lines. The mechanism may be related to suppression of the mTOR signaling pathway.

## Introduction

Neuroblastoma (NB) is the most well-known intracranial solid tumor in childhood, accounting for approximately 7–10% of all malignant tumors in children and is the most common extracranial solid tumor in children [1]. Multiple strategies, such as surgery, chemotherapy, radiotherapy, autologous stem cell transplantation, and various combinations of these therapies, have been used to treat NB [2]. However, even with a series of progressive adjuvant therapies, the prognosis in late childhood is still poor, especially for children older than 1 year, and the long-term survival rate is still below 40% [3]. This highlights the restrictions of existing treatment options and calls for more effective treatment strategies.

Autophagy, a process for resolving and recycling proteins and damaged cellular organs, has been proposed to protect cells from cellular stress or nutritional limitations and to regulate cell death pathways [4]. Multiple studies have closely linked autophagy to many disease processes, especially cancers [5]. The role of autophagy in cancer therapy is currently unclear. Some studies have shown that autophagy plays a significant role in cell survival in tumors [6,7]. However, recent research has found that autophagy plays a significant role in anticancer therapy, and the enhancement of autophagy tends to inhibit tumorigenesis [8–10]. Therefore, the induction of autophagy is currently considered a novel therapeutic method.

Rapamycin is a special prophylactic for the mammalian target of rapamycin (mTOR), which binds fk506-binding protein 12 kDa (FKBP12) to form a molecular complex that inhibits mTOR activity [11]. Abnormal activation of mTOR can lead to the occurrence of a wide variety of tumors [12]. Moreover, the promotion of autophagy linked to mTOR inhibition may mediate some effects of mTOR on cancer. mTOR signaling is one of the major pathways in the management of autophagy, and the implications of mTOR signaling in cancer have been thoroughly investigated over the last decade. However, the mechanism by which Rapamycin promotes autophagy to reduce proliferation has not been reported in NB.

In the present study, we focussed on the influence of Rapamycin on proliferation and autophagy in human NB cell lines. Furthermore, we analyzed the expression of autophagy-related proteins to study the role of Rapamycin in autophagy. Our results provide a new basis for the future treatment of NB.

## Materials and methods

### Cell culture and materials

The Institute of Cell Biology of the Chinese Academy of Sciences acquired the human NB cell lines SK-N-SH and SH-SY5Y. All cells were maintained in high-glucose Dulbecco’s modified Eagle’s medium (DMEM) (Gibco, U.S.A.), supplemented with 10% FBS (Gibco, U.S.A.), 100 units/ml penicillin, and 100 μg/ml streptomycin, in a humidified atmosphere of 5% carbon dioxide at 37°C. The medium was changed once every 2 days. Rapamycin powder was purchased from Sigma Chemical Co. (U.S.A.). All reagents were diluted in DMSO (Sigma, U.S.A.) and kept at −20°C. Antibodies included anti-Beclin-1 (ab114071, Abcam, U.K.), anti-LC3-I/II (ab114071, Abcam, U.K.), anti-P62 (ab91526, Abcam, U.K.), anti-mTOR (ab109268, Abcam, U.K.), anti-p-mTOR (ab32028, Abcam, U.K.), anti-GAPDH (ab8245, Abcam, U.K.), and goat anti-rabbit IgG-HRP (Santa Cruz Biotechnology, CA).

### Cell proliferation test

Cell proliferation assays were carried out using a cell counting kit-8 (CCK-8; Dojindo, Japan) in accordance with the manufacturer’s protocol. NB cell lines SK-N-SH and SH-SY5Y were seeded in 96-well plates (1 × 10^4^ cells/well). Following 12 h of incubation, 0.1 µl of Rapamycin was added to the wells at 10, 20, 30, and 40 µM, whereas 0.1 µl DMSO was used as the control group, single SK-N-SH cells as the negative group, and acellular group as the blank group. Following Rapamycin treatment for 12, 24, and 36 h, the culture medium was removed and 100 µl of DMEM was added, then 10 µl of CCK-8 was added to the cells, which were incubated for an additional 1 h at 37°C in the dark. Next, a microplate reader was used to measure the absorbance of each well at 450 nm, and inhibition rates were calculated as follows: Inhibition rate (%) = {[A450(NC) − A450(sample)]/[A450(NC) − A450(blank)]} × 100%.

### Flow cytometric evaluation of cell cycle

Cells were seeded into six-well plates at a density of 3 × 10^5^ cells/well. Cells were cultured with drug solution for 24 h, harvested with 0.25% trypsin (no EDTA), washed with 4°C PBS, immobilized with 75% ethyl alcohol at 4°C overnight, and then washed twice with 4°C PBS. Cells were then suspended in 100 μl of 5 mg/ml RNase solution, incubated at room temperature in the dark for 30 min, and then stained with 0.05 mg/ml Propidium Iodide (PI) solution for 20 min at 4°C in the dark, after which the stained cells were collected for analysis with a FACS Calibur Flow Cytometer (BD Accuri™ C6, CA).

### TEM

TEM was employed to visualize the occurrence of autophagy as evaluated by autophagosome formation. NB cell lines SK-N-SH and SH-SY5Y were counted and adjusted to a density of 1 × 10^6^ cells/ml and then fixed for 24 h at 4°C in 2.5% glutaraldehyde and another 2 h in 1% osmium tetroxide, followed by an increasing gradient dehydration step using ethanol and acetone. Cells were then embedded in epoxy resin and ultrathin sections were cut, and stained with 0.2% lead citrate and 1% uranyl acetate. Autophagosomes were observed by TEM (Wenzhou Medical University, H-7500, HITACHI, Japan) and imaged. Autophagosome structures were characterized by material surrounded by a double-layered membrane, with a higher electron density compared with the cytosol.

### Western blot analysis of Beclin-1, LC3-I/II, P62, mTOR, p-mTOR, and GAPDH

**Figure 1:**
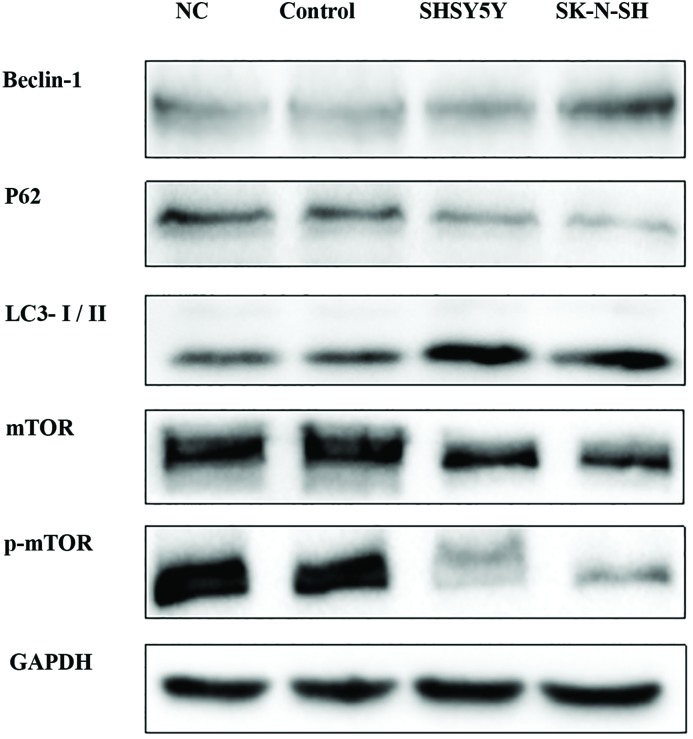
WB result: expression of Beclin-1, P62, LC3-I/II, mTOR, and p-mTOR were quantitated and observed by photograph imaging equipment

Autophagy-related proteins Beclin-1, LC3-I/II, and P62, and the mTOR pathway-related proteins mTOR, p-mTOR, and GAPDH were analyzed by Western blot. After treatment of NB cells with Rapamycin (20 μM) for 24 h, the cells were harvested and incubated in Total Histone Extraction Kit on ice for 10 min. Then the lysate was clarified by centrifugation at 12000***g*** for 10 min at 4°C to obtain the supernatant (total cell lysate). The total protein concentration was determined using the Coomassie Brilliant Blue (CBB) method. For Western blotting (WB), protein samples (100 μg/sample) were separated by SDS/PAGE and transferred on to PVDF transfer membranes. After blocking of non-specific binding sites with 5% non-fat dry milk for 2 h at room temperature, membranes were incubated at 4°C with primary antibodies to detect Beclin-1 (1:1000), LC3-I/II (1:1000), P62 (1:1000), mTOR (1:1000), p-mTOR (1:1000), and GAPDH (1:1000) overnight. After washing the membranes to remove unbound primary antibodies, they were incubated with either horseradish peroxidase–conjugated anti-rabbit secondary antibody (1:5000) for 1 h at room temperature. Finally the membranes were washed with TBST and chemiluminescence developed using ECL kit (Bio-Rad, Hercules, CA) for 1 min. Protein bands were visualized by image scanning and the optical density for each band was measured using Image Lab software (version 4.0, Bio-Rad, U.S.A.) after data were normalized to GAPDH as an internal control (Figure 1).

## Statistical analysis

Data were analyzed using SPSS 21.0 software and one-way ANOVA. The results are presented as the mean ± S.D. If homogeneity of variance was observed, the least significant difference (LSD) method was used to statistically analyze differences between groups. If differences were observed, we used Dunnett’s T3 test for statistical analysis. Differences with *P*-values less than 0.05 were considered statistically significant.

## Results

### Influence of Rapamycin treatment on NB cells proliferation

**Figure 2:**
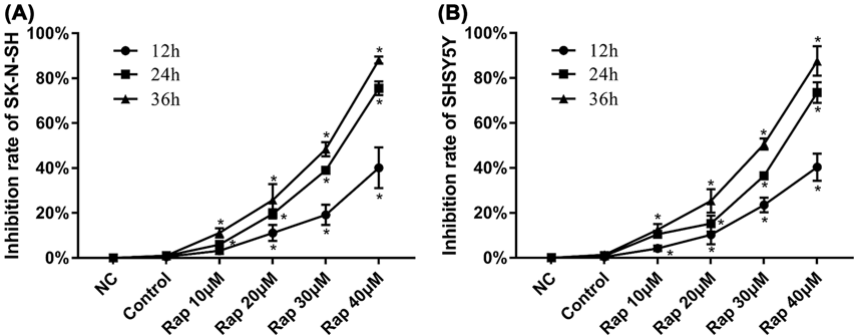
Effect of Rapamycin on the proliferation in NB cells. (**A**) Effect of Rapamycin treatment on SK-N-SH cell proliferation. (**B**) Effect of Rapamycin treatment on SH-SY5Y cell proliferation. Rapamycin, except treatment of 10 µM for 12 h, can obviously inhibited proliferation of NB cells compared with corresponding control group (*P*<0.05). (*) indicates statistically significant difference with *P*<0.05.

The influence of Rapamycin on cell proliferation was evaluated, and a suitable therapeutic concentration and treatment time were identified. NB cell lines SK-N-SH and SH-SY5Y were treated with 10, 20, 30, and 40 µM Rapamycin for 12, 24, and 36 h. Cells were then harvested, and cell proliferation was measured. As shown in Figure 2, Rapamycin inhibited the proliferation of NB cells in a dose- and time-dependent manner. On this basis, 24 h with 20 µm rapamycin were chosen as the duration and concentrations for drug treatment in subsequent experiments.

### Effect of Rapamycin treatment on NB cells cycle

**Figure 3:**
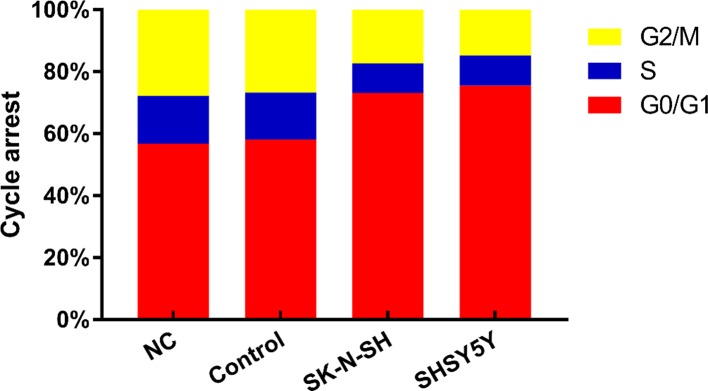
Cell-cycle analysis of NB cells for 24 h The percentage phase of G_0_/G_1_ increased in Rapamycin group while the percentage phase of G_2_/M decreased in Rapamycin group compared with control group (*P*<0.05).

Cell viability assays revealed that Rapamycin treatment significantly inhibited the proliferation of NB cells. Subsequently, whether the inhibitory effect on cell proliferation was related to cell cycle progression was assessed. As shown in Figure 3, flow cytometry (FCM) analysis indicated that 20 µM Rapamycin treatment for 24 h resulted in an increase in the proportion of NB cells in the G_0_/G_1_ phase compared with that in the control cells (*P*<0.05). Furthermore, the proportion of NB cells in the G_2_/M phase was greatly reduced after Rapamycin treatment compared with the control cells (*P*<0.05). These results indicated that Rapamycin can block the cell cycle at the G_0_/G_1_ stage in NB cells.

### Rapamycin induced formation of autophagosomes in NB cells

**Figure 4:**
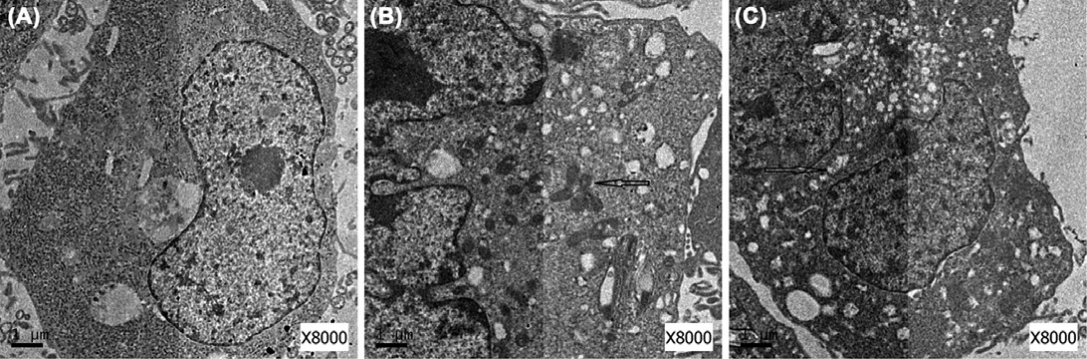
Morphological changes in NB cells. (**A**) Morphological changes of SK-N-SH cell in control group; (**B**) morphological changes of SK-N-SH cell in Rapamycin group; (**C**) morphological changes of SH-SY5Y cell in Rapamycin group.

The formation of autophagosomes in NB cells treated with 20 µM Rapamycin for 24 h was analyzed. As shown in Figure 4, in control cells, autophagic vacuoles were rare, while in Rapamycin-treated cells, there were many double-membrane autophagosomes and single-membrane autolysosomes.

### Rapamycin strengthened the expression of Beclin-1 and LC3-II/LC3-I and inhibited the expression of P62

**Figure 5:**
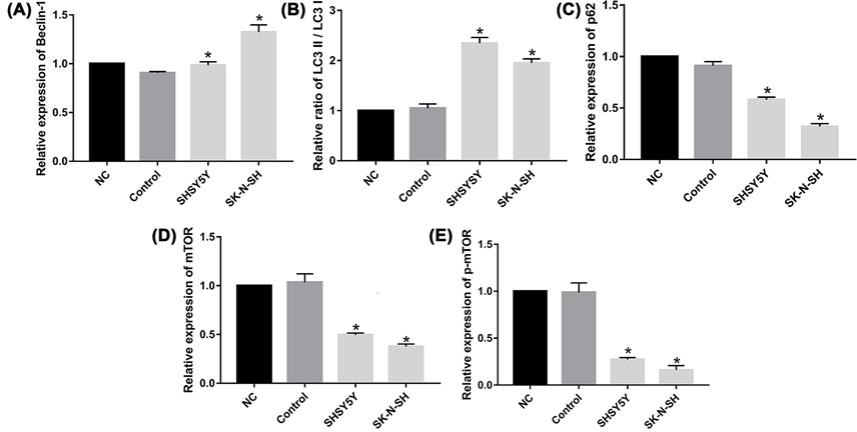
Rapamycin enhanced the expression of Beclin-1 and LC3-II/LC3-I while inhibited the expression of P62, mTOR and pmTOR All the results were representative of three independent experiments. (**A**) The expression of Beclin-1 was higher in Rapamycin group compared with control group (*P*<0.05). (**B**) The expression of LC3-II/LC3-I were increased in Rapamycin group (*P*<0.05). (**C**) The expression of P62 decreased in Rapamycin group compared with control group (*P*<0.05). (**D**) The expression of mTOR decreased in Rapamycin group compared with control group (*P*<0.05). (**E**) The expression of p-mTOR also decreased in Rapamycin group compared with control group (*P*<0.05). (*) indicates statistically significant difference with *P*<0.05.

Beclin-1, LC3-II/LC3-I, and P62 are classical autophagic markers, and Beclin-1 is required for the initiation of autophagosome formation. The expression of Beclin-1 and LC3-II/LC3-I was increased by Rapamycin treatment compared with the control cells (*P*<0.05) (Figure 5A,B). In contrast, P62 showed reduced expression in Rapamycin-treated cells compared with the control cells (*P*<0.05) (Figure 5C). All experiments were repeated three times, demonstrating that Rapamycin enhances the expression of Beclin-1 and LC3-II/LC3-I, while reducing the expression of P62 and promoting autophagosome formation.

### Rapamycin inhibited the expression of mTOR and p-mTOR

For a more in-depth analysis of the influence of Rapamycin on autophagy and the associated signaling pathway, we also examined the expression of related proteins by WB. We found that mTOR and p-mTOR levels were clearly reduced by Rapamycin treatment compared with the control cells (*P*<0.05) (Figure 5D,E). All experiments were repeated three times. Together, these data strongly show that Rapamycin induces autophagy via the mTOR pathway in NB cells.

## Discussion

NB is the most well-known and deadly solid tumor in children, with 1200 new cases per year diagnosed in the United States and Europe [13,14]. The majority of these patients have metastatic disease characterized by rapid overgrowth and diffuse invasion. Despite progress in the use of improved diagnostic methods and intensive multimodal treatments to improve the cure rate of other pediatric neoplasms, the survival rate for NB patients has lagged [15,16]. Therefore, it remains important for investigators to develop novel treatment strategies for NB.

Rapamycin is a macrocyclic lactone isolated from *Streptomyces hygroscopicus* [17]. It has been widely used in the clinic as an antiproliferative drug and immunosuppressant. Presently, more and more studies have focussed on the effects of Rapamycin in tumor therapy. The present research mainly studied the effect of Rapamycin in human NB cell lines. Cell proliferation experiments showed that Rapamycin curbed the proliferation of NB cells. We confirmed that this is the basis of Rapamycin’s antitumor ability and demonstrated that Rapamycin can play a synergistic role in tumor attenuation. Another significant characteristic of tumors is the loss of cell cycle regulation, which allows cancer cells to proliferate without limit. We examined the G_0_/G_1_, S, and G_2_/M stages in NB cells. Cell cycle analysis implied that Rapamycin arrested the cell cycle in the G_0_/G_1_ stage, thus inhibiting DNA replication and the proliferation of cancer cells.

Previous research revealed that Rapamycin prevented NB cell proliferation by down-regulating MYCN protein expression, which may be related to the PI3K/Akt/mTOR pathway [18–20]. However, studies of the mechanism underlying the inhibitory effect of Rapamycin on NB cell proliferation have seldom mentioned the interaction between Rapamycin and autophagy or the molecular pathways involved in this process. To investigate the function of Rapamycin-induced autophagy during the antiproliferative process in tumor cells, we quantitated the protein levels of autophagic markers in NB cells.

*Beclin-1* is the first mammalian gene found to mediate autophagy, such as regulating the turnover of proteins controlling the growth and proliferation of cells [21]. LC3 is now widely used as a marker to monitor autophagy. Moreover, the detection of LC3 conversion (LC3-I into LC3-II) by analyzing the ratio of LC3-II/LC3-I is more informative because the amount of LC3-II is correlated with the number of autophagosomes [22]. P62 is a selective substrate of autophagy, which delivers protein aggregates for autophagic degradation through its LC3-interacting region [23]. In this study, WB analysis indicated that Rapamycin increased Beclin-1 levels and the ratio of LC3-II/LC3-I but decreased P62 levels. Similarly, we observed an increase in autophagosome formation by EM in cells exposed to Rapamycin. We found many double-membrane autophagosomes and single-membrane autolysosomes within these treated cells. Inside autophagosomes, there was evidence of endoplasmic reticulum, mitochondria, and Golgi complex decomposition. Therefore, we confirmed that Rapamycin promotes autophagy in NB cells.

mTOR has been shown to play an important role in cell proliferation, metabolism, and tumor development, and proteins that modulate signals through mTOR are frequently changed in human cancers [20]. In addition, the mTOR signaling pathway is consistently activated in NB tumors [24]. Rapamycin is a well-studied inhibitor of mTOR, which specifically binds to mTOR and activates the autophagy of cells [25]. To better discriminate the role of mTOR protein in Rapamycin-triggered autophagy, we used WB to detect the expression of mTOR and p-mTOR. From the WB results, we found that Rapamycin increased LC3-II and Beclin-1 but decreased mTOR and p-mTOR. Therefore, these results imply that Rapamycin can inhibit the mTOR pathway to increase autophagy.

In summary, we determined that Rapamycin repressed the proliferation of human NB cell lines and induced autophagy by inhibiting the mTOR pathway. Our findings indicate that Rapamycin is a potential new target for further treatment of NB, and we predict that autophagy promoters that target mTOR will be a useful strategy for clinical treatment of NB.

## Abbreviations

CCK-8: cell counting kit-8
DMEM: Dulbecco’s modified Eagle’s medium
GAPDH: glyceraldehyde-3-phosphate dehydrogenase
HRP: horseradish peroxidase
mTOR: mammalian target of Rapamycin
LC 3: microtubule associated protein 1A/1B-light chain 3
MYCN: myelocytomatosis viral ralated oncogene neuroblastoma derived
NB: neuroblastoma
PI3K/Akt: phosphatidylinositol 3-kinase/protein kinase B
TBST: Tris buffered saline Tween
